# 3D-Printed Cell-Instructive Scaffolds Based on *Chondrosia reniformis* Collagen and Sr-Doped Calcium Phosphates for Bone Tissue Engineering

**DOI:** 10.1021/acsbiomaterials.4c01926

**Published:** 2025-05-06

**Authors:** Miguel S. Rocha, Catarina F. Marques, Sandra Pina, Joaquim M. Oliveira, Rui L. Reis, Tiago H. Silva

**Affiliations:** † 3B’s Research Group, I3Bs − Research Institute on Biomaterials, Biodegradables and Biomimetics, University of Minho, Headquarters of the European Institute of Excellence on Tissue Engineering and Regenerative Medicine, AvePark, Parque de Ciência e Tecnologia, Zona Industrial da Gandra, Barco, Guimarães 4805-017, Portugal; ‡ ICVS/3B’s−PT Government Associate Laboratory, Braga, Guimarães 4806-909, Portugal

**Keywords:** marine biomaterials, collagen, calcium phosphates, strontium, 3D printing, bone regeneration

## Abstract

Bone defects pose a global concern due to their high prevalence. Despite the significant advances in the development of novel therapies and sustainable biomaterial solutions, these still do not perfectly address the clinical needs, in particular, the paradigm shift of personalized treatments. In this sense, marine-origin materials allied to three-dimensional (3D) printing are arising as a feasible alternative to develop innovative personalized approaches, namely, bone tissue engineering (TE). In this study, novel 3D-printed scaffolds composed of collagen obtained from the maricultured marine sponge *Chondrosia reniformis* and calcium phosphates extracted from codfish (*Gadus morhua*) bones doped with strontium, and combined with alginate, were developed as a promising approach for bone regeneration. The 3D-printed scaffolds demonstrated suitable pore size and porosity and high interconnectivity, with adequate mechanical properties for bone TE. The *in vitro* assays conducted with a human osteosarcoma cell line (Saos-2 cells) cultured onto the 3D-printed scaffolds demonstrated a notable improvement in both cell viability and proliferation up to 14 days of culturing. This enhancement was particularly evident in the case of 3D-printed scaffolds containing Sr-doped calcium phosphates. Aligned with the principles of the blue economy and within a sustainable development approach, an innovative 3D-printed scaffold produced from sustainable marine-derived collagen and strontium-doped calcium phosphates with adequate mechanical properties, architecture, and encouraging *in vitro* performance was developed for bone tissue engineering scaffolding applications.

## Graphical abstract

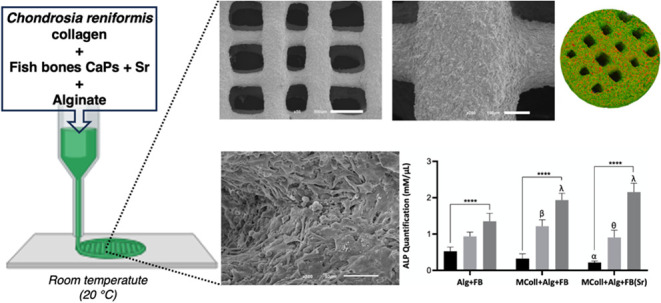

## 1 Introduction

Collagen has been extensively used to produce 3D structures for diverse tissue engineering and regenerative medicine (TERM) applications, ranging from skin to bone regeneration.^1^ It is the most abundant structural protein, extremely similar in both vertebrates and invertebrates, and it is mainly responsible for maintaining the structure of the extracellular matrix (ECM).^2^ Collagen has advantageous biological properties such as the presence of RGD (Arginine-Glycine-Aspartic acid) domains, which favor cell attachment, growth, and migration due to specific interactions with cell integrin receptors.^2^ Presently, the main industrial collagen sources are bovine and porcine byproducts, but risks of zoonosis and religious and social constraints have encouraged research for alternative collagen sources.^3,4^ In this sense, marine organisms have gained wide acceptability as safe and sustainable sources for diverse applications, such as pharmaceutical, cosmetic, and TERM.^5^

*Chondrosia reniformis* is a marine sponge rich in collagen studied as a promising sustainable collagen source due to its great mariculture potential.^6^ Porifera collagens are known to be more glycosylated than other collagens in Metazoa, and the high abundance of glycosaminoglycans favors cell attachment and proliferation, enhancing their regenerative potential.^7^ Additionally, *C. reniformis* ectosome-derived collagen (MColl) has been described as presenting cytocompatibility and possessing similarities with collagen type I and IV, the former beneficial for bone regeneration strategies.^8^

Furthermore, *C. reniformis* collagen has been previously used to develop microparticles and membranes,^9,10^ but it has yet to be successfully employed as an ink in additive manufacturing (AM), particularly 3D printing. This processing technique allows for better control of the scaffolds’ architectural features, such as pore size and porosity, as compared to conventional scaffolding methodologies, including freeze-drying and salt-leaching processes.^11^ There are seven classifications of 3D printing processes for the fabrication of cell-instructive scaffolds, each with its benefits and drawbacks, thus making it essential to choose the right fabrication techniques for the intended application.^12^ In TERM, the most commonly used have been extrusion, stereolithography, laser-assisted, inkjet, and microvalve-based printers.^12^ Material extrusion is the most conventional 3D printing technique, extensively used for the fabrication of 3D-printed medical devices. Furthermore, extrusion-based 3D printing is mainly chosen for printing with natural or synthetic materials combined with insoluble particles, such as calcium phosphates (CaPs),^13^ often included to improve the properties of the resulting inks.

Currently, there is a scientific and technological demand regarding the development of new bioinspired ink formulations for biomedical applications, which must present bioactivity, biocompatibility, and printability. The design of 3D-printed scaffolds using collagen-based inks is limited by the printability requirement, which is challenging considering the collagen’s low mechanical strength, and the influence of the environmental conditions, as temperature could modify collagen viscosity.^14^ To overcome this challenge, a biodegradable polymer, such as alginate (Alg), can be added to the ink’s formulation, which is characterized by the ability to rapidly *in situ* cross-link in the presence of di- or trivalent ions (e.g., Ca^2+^).^15^ Alternatively, or in complement, the combination with calcium phosphates has also been explored, with the addition of this ceramic being reported to reinforce collagen-based matrices.^16^

Moreover, calcium phosphates (CaPs) are biocompatible materials holding great potential for biomedical applications,^17^ which can be extracted from natural sources such as fish bones (FB). By means of using an industrial marine byproduct such as codfish (*Gadus morhua*) bones (FB) for the production of CaPs, an added-value product is developed from what is mostly considered waste by the fish-processing industry, thus demonstrating its great potential.^18^ Single (hydroxyapatite (HAp)) or biphasic (HAp and β-tricalcium phosphate (β-TCP)) phases can be obtained, depending on the processing conditions and content of the fish bone.^19^ However, biphasic CaPs possess superior performance when compared to single phases,^20^ by combining the high mechanical properties of HAp with the fast bioresorbability of β-TCP, thus allowing to control of the resorption rate of these biomaterials and leading to more intricate biological and chemical events.^21^ Furthermore, it has been demonstrated that adding trace elements to CaPs, by partially substituting Ca^2+^ with other ions, can enhance the mechanical properties and the biological response of the biomaterials.^22^ For instance, strontium (Sr^2+^) has been shown to depress bone resorption, while maintaining bone formation by increasing osteoclast apoptosis and stimulating cell pre-osteoblast proliferation.^23^ Consequently, it was hypothesized that adding Sr-doped CaPs to the ink formulation would not only enhance the mechanical properties but also improve the biological activity of the 3D-printed scaffolds.

Therefore, we propose for the first time the production of 3D-printed scaffolds composed of *C. reniformis* collagen combined with Alg, and CaPs extracted from codfish bones (FB) doped with Sr (FB­(Sr)), aiming at bone regeneration. Bone composition was taken into account when considering the inks’ formulations for scaffolds fabrication; therefore, a blending ratio of biopolymers 30 wt% MColl and Alg, and 70 wt% of FBs was established.^24^ The inks and 3D-printed scaffolds were extensively characterized, addressing their microstructure and morphology together with rheological and mechanical properties. Additionally, the proliferation and differentiation responses of osteoblast-like cells (Saos-2 human osteosarcoma cell line) cultured on the scaffolds were examined, including viability, DNA quantification, and alkaline phosphatase (ALP) activity, as *in vitro* model for the assessment of bone tissue formation.

## 2 Materials and Methods

### 2.1 Materials and Reagents

Specimens of *C. reniformis* from mariculture trials performed at Pina Reef in Kas-Kekova Special Environmental Protected Area, Turkey, were collected by Scuba diving at depths between 5 and 20 m by partners of Wageningen University & Research, The Netherlands. Upon collection, specimens were frozen in ice and transported in dry ice containers by air transport to the facilities of 3B’s Research Group, Portugal, where they were kept at −20 °C until needed Codfish (*Gadus morhua*) bones (FB) were provided by the company Soguima (Guimarães, Portugal). All reagents were obtained from Sigma-Aldrich (St. Louis, MO), unless otherwise specified.

### 2.2 *C. reniformis* Collagen (MColl) Extraction

MColl extraction was performed as described elsewhere.^25^ In brief, marine sponge samples were thawed, and exogenous materials were eliminated by rinsing them with distilled H_2_O. Ectosome was separated from the choanosome and cut into minor pieces of roughly (1 × 1 × 1) mm^3^. Then disaggregating solution (50 mM Tris–HCl buffer pH 7.4, 1 M NaCl, 50 mM EDTA, and 100 mM 2-mercaptoethanol) was added to the ectosome and left to stir for 5 days. The resulting collagen suspension (CS) was filtered through a nylon mesh to remove any undissolved fragments and, then, extensively dialyzed using a 14 kDa cutoff dialysis cellulose membrane tubing for 7 days, with 2 buffer changes per day (CS/dialyzing buffer ratio 1:1000) against dH_2_O, to remove all 2-mercaptoethanol traces. The suspension was centrifuged (5810 R, Eppendorf, Hamburg, Germany) for 10 min at 1200*g* to eliminate cell debris and sand particles, followed by 30 min at 12,100*g* to collect the collagen from the suspension. Collagen re-extraction was carried out by repeating the second centrifugation step. All collagen extraction steps were performed at 4 °C. The collagen suspension obtained was freeze-dried and stored at room temperature (RT) until further use. The produced collagen, as previously reported in ref 8, had a high purity degree, with preserved triple helix and fibrillar conformation, possessed features of collagen types I and IV, but was insoluble in acidic solutions. Moreover, it was shown to be noncytotoxic for L929 cells, thus being well-suited for tissue engineering applications. A more detailed characterization study can be found in the mentioned ref 8

### 2.3 Preparation of CaPs Powders Doped with Strontium

First, CaPs powders were extracted from codfish (*G. morhua*) bones (FB). For that, FB was boiled in water for 1 h, washed to remove remaining pieces of adherent fish meat and organic substances, and then dried at 45 °C overnight in a vacuum oven. The dried FB were milled (Ultra Centrifugal Mill ZM 200, RETSCH, Haan, Germany) and sieved with a 1 mm mesh, followed by calcination in a furnace (Termolab, Aveiro, Portugal) using a heating rate of 5 °C/min up to 1000 °C and 2 h dwelling time at that temperature. Sr-doped FB (FB­(Sr)) powders were obtained by mixing concentrated aqueous suspensions (40 vol %) of FB with 10 mol% of strontium nitrate and 0.4 wt% of ammonium polycarbonate dispersant (Targon 1128), relative to the dry mass of solids. The suspension was left stirring for 3 h at RT until complete homogenization and then dried at 100 °C overnight in a vacuum oven. The FB­(Sr) powders were calcinated at 1100 °C, with a heating rate of 5 °C/min followed by a dwelling time of 12 h, and the calcinated powder was then passed through a 36 μm sieve. Pure FB was also used for comparison purposes.

### 2.4 Characterization of CaPs Extracted from Fish Bones

#### 2.4.1 X-Ray Diffraction (XRD)

The qualitative and quantitative analysis of phase crystallinity of CaPs powders was conducted using a high-resolution Bragg–Brentano X-ray diffractometer (D8 Advance DaVinci, Bruker, Bremen, Germany) equipped with Cu Kα radiation (λ = 1.5406 Å) generated at 40 kV and 40 mA. Data sets were collected in the 2θ range of 10–60° with a step size of 0.02° and 1 s per step. The phase composition of the powders was determined from the XRD patterns by using Rietveld analysis with TOPAS 5.0 software (Bruker, Bremen, Germany). The refined parameters included the scale factor, sample displacement, background as a Chebyshev polynomial of fifth order, crystallite size, and lattice parameters.

#### 2.4.2 Fourier Transform Infrared in Attenuated Total Reflection Mode (FTIR-ATR)

Infrared spectra of the powders were analyzed by FTIR under ATR. FTIR-ATR measurements were conducted by using an IR-Prestige-21 spectrophotometer (Shimadzu Scientific Instruments, Columbia, MD). Each infrared spectrum was the average of 32 scans collected at a resolution of 2 cm^–1^ in the wavenumber region of 3600–500 cm^–1^ at RT.

#### 2.4.3 Scanning Electron Microscopy (SEM)

The microstructure of the Sr-doped CaPs powders was observed by SEM (JSM-6010 LV, JEOL, Tokyo, Japan), using a beam energy of 10 keV at different magnification levels. The powders were sputter-coated with gold using the Leica EM ACE600 sputter coater (Leica, Wetzlar, Germany), before the SEM analysis.

### 2.5 Production of 3D-Printed Scaffolds

#### 2.5.1 Inks Optimization and Characterization

The inks for 3D printing were prepared using different formulations of MColl and Alg and then combined with pure and Sr-doped FB, employing a blending ratio of 30 and 70 wt %, respectively. MColl solutions of 1, 2, and 5% (w/v) and Alg solutions of 5, 8, and 10% (w/v) were prepared by solubilization in water and left overnight under magnetic stirring. After performing pilot studies testing different combinations to identify the formulation with the best printing fidelity, MColl and Alg concentrations were fixed at 5 and 10%, respectively, and combined in an MColl/Alg ratio of 2:1 (v/v). After dissolution of the polymers, FB and FB­(Sr) powders were added with a high solid volume concentration, to minimize shrinkage during drying, so that the particle network could withstand compressive stresses generated by capillary tension, and thoroughly mixed.^26^ Then, the polymer/FB mixtures were cross-linked with 150 μL of 6% (w/v) CaCl_2_ solution. Three different formulations were prepared: Alg with FBs (Alg + FB), MColl + Alg with FB (MColl + Alg + FB), and MColl + Alg with FB­(Sr) (MColl + Alg + FB­(Sr)), as depicted in Table 1. To assess the printability of the different inks’ composition, a rheometer (Kinexus Prot, Malvern Instruments, Worcestershire, U.K.) was used under oscillatory and viscometry conditions. The apparent viscosity was assessed in viscometry mode using a cone and plate sensor system (4°/40 mm; CP4/40 SR1772SS) with a shear rate from 0.01 to 100 s^–1^. The viscoelastic properties were assessed by equipping the rheometer with a stainless steel parallel plate of 8 mm diameter (PU20 SR1740SS) and a plate sensor with a 1 mm gap size. For the temperature sweep, the temperature ranged between 4 and 45 °C with a ramping rate of 2 °C min^–1^. For all measurements, a frequency of 1 Hz and shear stress values within the linear viscoelastic region (LVR) were chosen. Frequency sweeps were also performed on each sample, acquiring 10 points per decade from 0.01 to 100 Hz to determine the elastic modulus (*G*′), loss modulus (*G*″), and viscosity. All plots were generated by averaging the results of at least 3 experiments.

**Table 1:** Formulations of the Inks Developed for 3D Printing

formulation	alginate (Alg) concentration	collagen (MColl) concentration	fish bone CaPs (FB)	Sr^2+^ doped fish bone CaPs (FB(Sr))	polymers/CaPs ratio
Alg + FB	10%		√□		30:70
MColl + Alg + FB	10%	5%	√□		30:70
MColl + Alg + FB(Sr)	10%	5%		√□	30:70

#### 2.5.2 3D Printing of the Scaffolds

The inks were centrifuged for 2 min at 1000*g* to remove air bubbles and then carefully transferred into the cartridges to minimize the formation of new bubbles. Each cartridge was filled with 3 mL of ink, followed by the 3D printing process. The 3D printing process was carried out at RT using a bioprinter V1 (REGEMAT 3D, Granada, Spain) with a printing nozzle of 410 μm of inner diameter at a printing speed of 10 mm/second. A standard 3D printing software was utilized to design the 3D mesh structures: 7 × 7 × 2 mm^3^ (width × length × height), in a total of 6 layers with 600 μm of pore size. After printing, the 3D structures were ionically cross-linked with a 6% (w/v) CaCl_2_ solution overnight. Subsequently, the material was punched into 6 mm diameter cylindrical scaffolds and freeze-dried.

### 2.6 3D-Printed Scaffolds Physicochemical Characterization

#### 2.6.1 Scanning Electron Microscopy with Energy-Dispersive Spectroscopy (SEM-EDS)

To evaluate the microstructure and surface morphology, the 3D-printed scaffolds were observed by SEM (JSM-6010 LV, JEOL, Tokyo, Japan), using a beam energy of 10 keV at different magnification levels. The scaffolds were freeze-dried and then sputter-coated with gold using the Leica EM ACE600 sputter coater (Leica, Wetzlar, Germany) before analysis. The energy-dispersive X-ray spectroscopy (EDS) was used to characterize the elemental chemical composition of the scaffolds. The EDS analyses were performed using the INCAx-Act, PentaFET Precision (Oxford Instruments, Abingdon, U.K.) instrument at an energy of 10.0 keV coupled to SEM.

#### 2.6.2 Microcomputed Tomography (μCT)

The morphological and morphometric properties of the scaffolds, as well as the distribution of the biomaterials throughout the scaffolds, were evaluated by a high-resolution μCT SkyScan 1217 (Bruker, Bremen, Germany). The scanning of the scaffolds was conducted using an X-ray source fixed at 50 keV and 200 μA. Representative data were reconstructed using nRecon software (Bruker, Bremen, Germany), while CT Analyzer software (CT Analyzer v1.17, SkyScan, Kontich, Belgium) was employed to reslice all of the files of each sample and calculate various quantitative morphometric parameters, including porosity, pore size, and pore interconnectivity. Both 2D and 3D qualitative visualizations of the different phases at the scaffolds were obtained with Data Viewer (v1.7.1.0) and CTVox (v3.3.0.r1412) (SkyScan, Kontich, Belgium). Each of the parameters was measured in triplicate, and the data were presented as the mean ± standard deviation.

#### 2.6.3 Mechanical Testing

The mechanical properties of the printed scaffolds (Ø6 × 2 mm^3^) were evaluated using a universal material testing machine (Instron 5540, Norwood, MA) in compressive mode with a load cell of 1 kN. Seven replicates of each condition were tested at a crosshead speed of 1 mm/min until 20% deformation. Before each test, the compression plate was slowly and carefully lowered until it made contact with the top surface of the sample. The elastic modulus was calculated from the slope of the linear region of the stress–strain curve using Bluehill Universal software (Instron, Norwood, MA). The results were presented as mean ± standard deviation.

### 2.7 *In Vitro* Assessment of 3D-Printed Scaffolds

#### 2.7.1 Saos-2 Cell Culture and Seeding

Human osteosarcoma cell line (Saos-2 cells, ATCC HTB-85) was used to evaluate the cytotoxicity of the scaffolds. The Saos-2 cells were cultured in Dulbecco’s Modified Eagle’s Medium (DMEM) low glucose supplemented with sodium bicarbonate (3.7 g/L), 10% fetal bovine serum (FBS) (Thermo Fisher Scientific, Waltham, MA), and 1% Penicillin/Streptomycin (Thermo Fisher Scientific, Waltham, MA) and in a humidified controlled environment (37 °C, 5% CO_2_). Before confluence, cells were trypsinized using TrypLE Express (Thermo Fisher Scientific, Waltham, MA), and 3 × 10^5^ cells were seeded in the 3D-printed scaffolds. Briefly, 30 μL of culture medium containing the respective number of cells was cultured with the 3D-printed scaffolds and kept in a humidified controlled environment (37 °C, 5% CO_2_) for up to 14 days. Prior to cell seeding, sterilization of the 3D-printed scaffolds was performed by immersing the structures in cell culture medium supplemented with 10% Penicillin/Streptomycin (Thermo Fisher Scientific, Waltham, MA) for 10 min to avoid microbial contamination.^27^

#### 2.7.2 Metabolic Activity

The metabolic activity of cells was assessed at culture periods of 1, 7, and 14 days using the Alamar blue assay. At the indicated time points, the cells were incubated for 3 h at 37 °C with fresh medium containing 10% of Alamar blue solution (Thermo Fisher Scientific, Waltham, MA). Following, resazurin reduction from nonfluorescent blue to red fluorescent was spectrophotometrically measured using a microplate reader (SINERGY HT, BIO-TEK, Winooski, VT) at 530 nm excitation and 590 nm emission. The results were expressed as the mean ± standard deviation of three independent experiments (*n* = 3) with three replicates per condition.

#### 2.7.3 Cell Proliferation Assessment

Cell proliferation was assessed using a fluorometric double-stranded DNA (dsDNA) kit (Quant-IT PicoGreen dsDNA Assay Kit) according to the manufacturer’s instructions. The scaffolds were washed with Tris-buffered saline, and then, 1 mL of ultrapure water was added and maintained at 37 °C, followed by a thermal shock (−80 °C) to promote cell lysis and release of DNA to the medium. The fluorescence intensity of the scaffolds was measured using a microplate reader at 485:528 nm, and DNA content was determined using a standard curve generated with concentrations ranging from 0 to 2 μg/mL. The results were presented as the mean ± standard deviation of three independent experiments (*n* = 3) with three replicates per condition.

#### 2.7.4 Alkaline Phosphatase Activity Quantification

Alkaline phosphatase (ALP) activity was assessed in the different 3D-printed scaffolds using the cell lysates previously produced. For that, 80 μL of lysate was combined with 20 μL of 1.5 M alkaline buffer solution and 100 μL of 4 mg/mL of phosphatase substrate. The samples were incubated for 1 h at 37 °C, after which the reaction was stopped by using 0.3 N of NaOH, and the absorbance was read at 405 nm. The ALP standard curves were generated using various dilutions of a 10 mM 4-Nitrophenol solution. The results were presented as the mean of three independent experiments (*n* = 3) with three replicates per condition.

#### 2.7.5 Live/Dead Assay

Viability of the Saos-2 cells seeded on the scaffolds after 1, 7, and 14 days was performed using a live/dead cell viability assay. At the end of each culture period, the culture medium was removed, and calcein-AM and propidium iodide (PI) at final concentrations of 1 and 5 μg/mL in culture medium, respectively, were added to cell-seeded scaffolds. After 30 min at 37 °C in the CO_2_ incubator, the scaffolds were immediately observed in a Transmitted and Reflected Light Microscope (Carl Zeiss, Jena, Germany) with the green staining indicating the live cells and the red staining corresponding to dead cells.

#### 2.7.6 Scanning Electron Microscope (SEM)

To further evaluate cell number and morphology, 3D-printed scaffolds retrieved at specific time points were fixed in 4% paraformaldehyde (PFA), washed with Tris-buffered saline, dehydrated with increasing concentrations of ethanol (50 to 100%), incubated with hexamethyldisilazane (HMDS) overnight, and then sputter-coated with gold using the Leica EM ACE600 sputter coater (Leica, Wetzlar, Germany). The samples were then observed by analytical SEM (JSM-6010 LV, JEOL, Japan) at several magnifications.

### 2.8 Statistical Analysis

Data were presented as mean ± standard deviation (SD) of at least three independent assays. Statistical analyses were conducted using GraphPad Prism 8.0.1 (La Jolla, CA). Data normality was assessed using the Shapiro-Wilk test. For group comparison, a two-way ANOVA test was performed, followed by Tukey’s test to assess differences between the groups with a confidence level of 95%. The significance level between groups was represented by the following symbols: * (*p* < 0.05), ** (*p* < 0.01), *** (*p* < 0.001), and **** (*p* < 0.0001).

## 3 Results and Discussion

### 3.1 Pure and Sr-Doped Fish Bone Powders

The first goal of this study was the development of an ink composed by marine-derived materials obtained from sustainable sources, namely *C. reniformis* collagen and fish bone-derived calcium phosphates, for 3D printing of composite scaffolds,^16,28^ targeting bone regeneration.

Transforming an abundant byproduct, such as codfish bones, into a high-value material as CaPs is appealing in terms of both waste management and the production of advanced material. The inclusion of CaPs derived from a marine byproduct was proposed, taking into consideration environmental concerns, as well as the exceptional properties exhibited by these materials for bone tissue applications, namely improved biocompatibility and osteogenic differentiation activity.^17^ Moreover, it was hypothesized that the doping of FB with Sr would increase the biological performance and the bone tissue regeneration potential of the developed 3D-printed scaffolds

The effect of Sr-doping on the crystalline phase assemblage was investigated by using XRD (Figure 1A). It was possible to confirm that all of the diffraction peaks for the powders were assigned to calcium phosphate fluoride (CaP-F) apatite (ICDD card number of #01–083–7829 ^29^) and whitlockite (ICDD card number of #04–011–6758 ^30^) phases.^19,31^ All of the patterns showed the main peaks of CaP-F apatite at 31.9, 32.2, and 33.1°, as well as the main peak of whitlockite at 31.2°, thus indicating that the crystalline structure of apatite was maintained and no other phase was formed.^32^ The Rietveld refinement analysis confirmed the presence of CaP-F as the main phase with 89.7% and whitlockite with 10.3% in FB­(Sr). Furthermore, a detailed observation enabled the detection of slight shifts in the peak positions toward lower 2θ of the doped FB­(Sr) in comparison to undoped FB. Depending on the larger or smaller ionic radius of the dopant, the shifts were toward the left or the right, respectively.^33^ These results were in good agreement with other reports that showed the formation of a nonapatite phase (whitlockite) upon calcining fish bones with a low Ca/P atomic ratio.^34,35^

**Figure 1:**
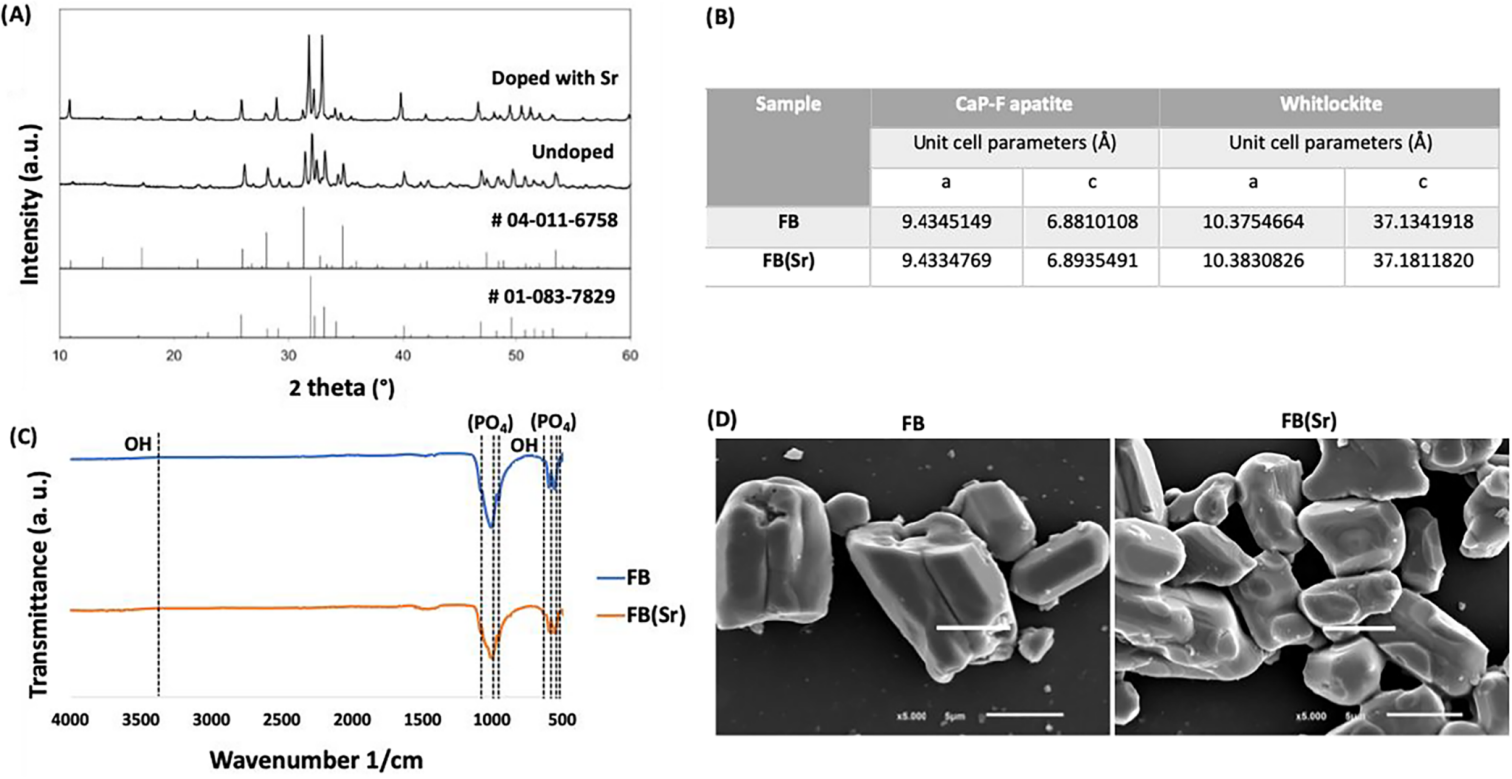
Undoped fish bones (FB) and Sr-doped fish bones [FB­(Sr)] physicochemical characterization. (A) X-ray diffraction (XRD) patterns of FB and FB­(Sr). The standards of CaP-F apatite and whitlockite are presented for comparison. (B) Refined lattice parameters for CaP-F apatite and whitlockite structures for FB and FB­(Sr). (C) Fourier transform infrared spectroscopy—attenuated total reflectance (FTIR-ATR) spectra of FB (blue line) and FB­(Sr) (orange line). (D) Scanning electron microscope (SEM) micrographs of FB and FB­(Sr).

Refined lattice parameters for the undoped and Sr-doped FB powders are presented in Figure 1B. The successful incorporation of Sr into the powder structure was further proven by the increment of the lattice parameters due to the higher ionic radii of Sr (1.12 Å) in comparison to Ca (0.99 Å), making the lattice expand. In addition, it was also possible to observe that the doping procedure did not affect the crystallinity of the powders, as the diffraction peaks were sharp and well-defined, which is characteristic of a crystalline material. Interestingly, the obtained patterns were in line with the ones acquired in Sr-doping procedures of other marine-based CaPs, namely, obtained from the conversion of ceramics retrieved from clam and crab shells.^36,37^

FTIR-ATR measurements performed on FB and FB­(Sr) powders are displayed in Figure 1C. All of the detected peaks of FB and FB­(Sr) were characteristic vibrational modes of CaP-F apatite phase formation.^33^ The bands at 3570 and 630 cm^–1^ were assigned to hydroxyl (–HO) group and those at 475, 575, 609, 966, and 1020–1120 cm^–1^ were assigned to phosphate (–PO_4_) groups of the apatite crystal, in accordance with previous works.^19,31,38^ The O–P–O bending mode (567–603 cm^–1^) and P–O bond stretching mode (960–1095 cm^–1^) were also detected.^19,39^ The presence of β-TCP was confirmed by the absorbance bands at 940 and 970 cm^–1^, which were due to ν1 nondegenerate P–O symmetry stretching mode, and the 1041 and 1160 cm^–1^ denoted triply degenerate ν3 antisymmetric vibration modes.^19,39^ Comparing the FTIR spectra from the undoped and doped FB, it was possible to observe an attenuation of the OH-related bands for the doped powder, which is in accordance with the literature.^31,36^ However, the spectra of non- and Sr-doped CaPs isolated from crab shells obtained by Handa and colleagues was slightly different, as there was a band of atmospheric carbon dioxide at 2360 cm^–1^ and a wide band (3200–3600 cm^–1^) associated with water molecules embedded into the structure of the CaP and CaP-Sr samples.^37^ Additionally, some fish-derived hydroxyapatite characterized by others^40,41^ presented peaks around 1650 cm^–1^ and in the region between 2826 and 3100 cm^–1^, corresponding to H–OH bending vibrations of adsorbed water and v­(CH) bands attributed to organic matrix, respectively, which were not found in our results, thus indicating the full removal of the organic content from the fish bone by the calcination method used in this work.

The SEM micrographs of FB and FB­(Sr) enabled to evaluate the powders’ morphologic features (Figure 1D), which presented crystals with a flake-like shape very similar to perch and salmon bone-derived hydroxyapatite powder.^42,43^ Particle size was found to fall within the range of a few micrometers, and the presence of an apparent agglomeration of the particles was observed, which was also observed in other fish-derived CaPs.^41,43^ The agglomeration may have been due to the strong capillary forces between the particles upon drying.^33^ Interestingly, the FB and FB­(Sr) morphology was distinct from the rod-like, elongated and dandelion-like structures attained in other works.^36,44–46^

### 3.2 Inks Characterization

To successfully develop 3D-printed scaffolds for biomedical applications, various challenges must be overcome, namely, obtaining inks with suitable viscoelastic properties for printing and adequate mechanical properties to follow tissue remodeling and regeneration.

Therefore, different inks composed of MColl (1, 2 and 5% w/v) and Alg (5, 8, and 10% w/v) concentrations, at different ratios of MColl/Alg (v/v) (1:1 and 2:1), were considered based on previous reports.^26,47^ A step-by-step optimization aiming to use the least alginate possible was used. In pilot studies, it was observed that it was not possible to print using only collagen, as the solution was overly fluid, an issue still occurring using lower collagen concentrations (1 and 2%) mixed with alginate (data not shown). However, appropriate printability was achieved by employing higher concentrations of both collagen (5%) and alginate (10%) combined at a ratio of 2:1, respectively. Therefore, MColl5% + Alg10% w/v (2:1 v/v) [with FB or FB­(Sr)], cross-linked with CaCl_2_ 6% (w/v), were selected since they showed the best printing fidelity and stability [inks MColl + Alg + FB and MColl + Alg + FB­(Sr)]. Alg 10% w/v (with FB) cross-linked with CaCl_2_ 6% (w/v) (ink Alg + FB) was used as a control.

The rheological properties of the different formulations were investigated by performing oscillatory (frequency and temperature) and viscometric tests (Figure 2). Both the storage modulus (*G*′) and the viscosity of the Alg + FB composition exhibited the highest values, followed by MColl + Alg + FB­(Sr) and MColl + Alg + FB. Considering the collagen-based inks, oscillatory (frequency and temperature) and viscometry tests showed that the MColl + Alg + FB­(Sr) ink had higher storage modulus (*G*′) and viscosity than the MColl + Alg + FB ink. Nevertheless, all formulations presented a *G*′ higher than the loss modulus (*G*″), typical of viscoelastic solids, in which the material has a predominant solid response due to the existence of an internal 3D network (Figure 2A). These results were attributed to the major effect of the Ca^2+^ ionic cross-linking on the higher amount of Alg present in the Alg + FB ink when compared with the collagen-containing inks.^48^ An important property of Alg is its ability to interact and form a gel when in contact with divalent cations (e.g., Ca^2+^ and Sr^2+^), yielding stable physically cross-linked structures through electrostatic interactions between the positive charge of the divalent cation with the negatively charged carboxylate group of *G* units within Alg that produced an egg box.^49^ Furthermore, as Sr^2+^ was not completely incorporated into the CaPs structure, there was an increment in the available cations, which was associated with an increase in Alg ionic cross-linking and, therefore, a higher *G*′ and viscosity observed on the MColl + Alg + FB­(Sr) ink. Notably, the MColl + Alg + FB­(Sr) ink presented a higher *G*′ than bioinks based on another marine collagen developed for the engineering of hard tissue.^26^ Moreover, even though Alg + FB ink presented the highest viscosity, all inks displayed viscosities similar to other developed marine collagen-based inks for the regeneration of hard tissues with shear thinning behavior (Figure 2B).^26^ This rheological behavior was required to maintain the 3D printing accuracy when using extrusion-based dispensing systems.^50,51^

**Figure 2:**
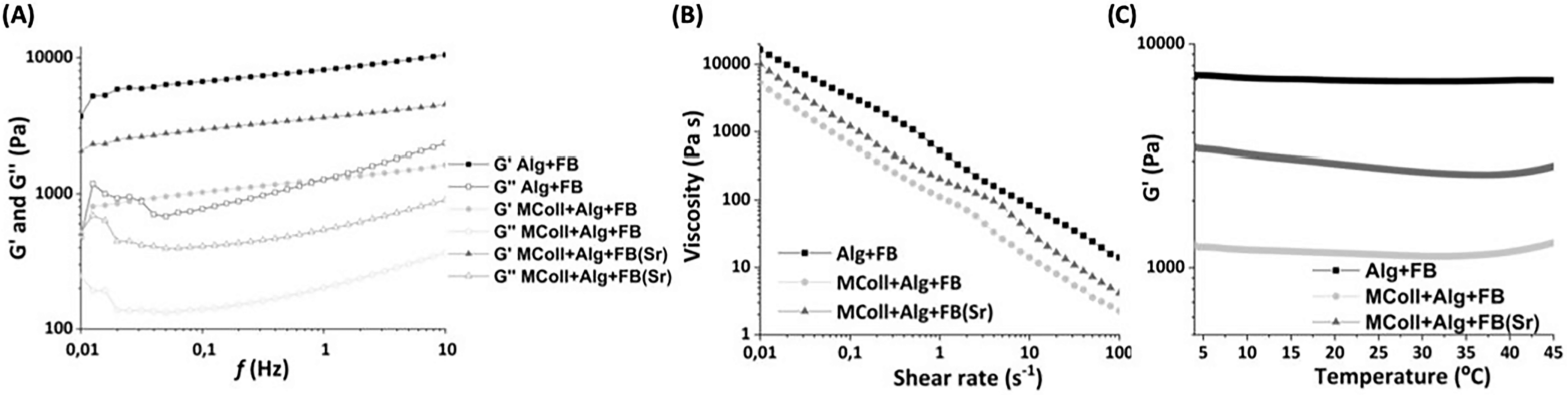
Rheological properties of the developed inks. (A) Storage modulus (*G*′) and loss modulus (*G*″) as a function of frequency. (B) Viscometry test: viscosity depending on the shear rate. (C) Oscillatory temperature test: storage modulus (G′) at each applied temperature.

Additionally, it was possible to observe that, as temperature increased, *G*′ was maintained relatively stable in all formulations (Figure 2C). In fact, at 37 °C, all inks herein developed preserved a *G*′ modulus that allowed the scaffolds’ structural integrity and printing fidelity. This behavior was beneficial since marine collagens tend to show lower thermal stability than mammal counterparts^47^ and has been demonstrated that porcine type I collagen exhibited the highest *G*′ at 41.5 °C.^52^

### 3.3 3D-Printed Scaffolds Characterization

As previously stated, the development of 3D-printed scaffolds using collagen-based inks is demanding mainly due to the low mechanical properties of collagen. Therefore, to ensure that the 3D printing process was reliable and reproducible, thorough characterization of the obtained 3D-printed scaffolds was performed.

The microstructure of the 3D-printed scaffolds observed by SEM and respective elemental analysis using EDS are presented in Figure 3B. Uniform filaments and well-defined shapes could be observed while maintaining the scaffolds’ structure after printing. Although the MColl + Alg + FB scaffolds exhibited microscopic fissures, which could be artifacts due to the freeze-drying process required for SEM analysis, their structural stability does not seem to have been adversely affected. From EDS spectra, it was possible to detect the presence of C, O, Ca, and P, associated with the organic and inorganic components of the ink, respectively, but the identification of Sr occurred only in the MColl + Alg + FB­(Sr) composition, which is in agreement with the established formulations and the obtained XRD and FTIR-ATR results. Furthermore, the printing accuracy achieved was comparable to that of studies employing collagen derived from mammalian sources.^52,53^ It was well reported that employing a low temperature (−40 °C) platform, the collagen:alginate-based inks printability was improved,^54^ while the use of covalent cross-linking increased the collagen stability.^55^ Remarkably, in the present work, the printing fidelity was achieved with RT printing conditions and without any chemical cross-linking agent.

**Figure 3:**
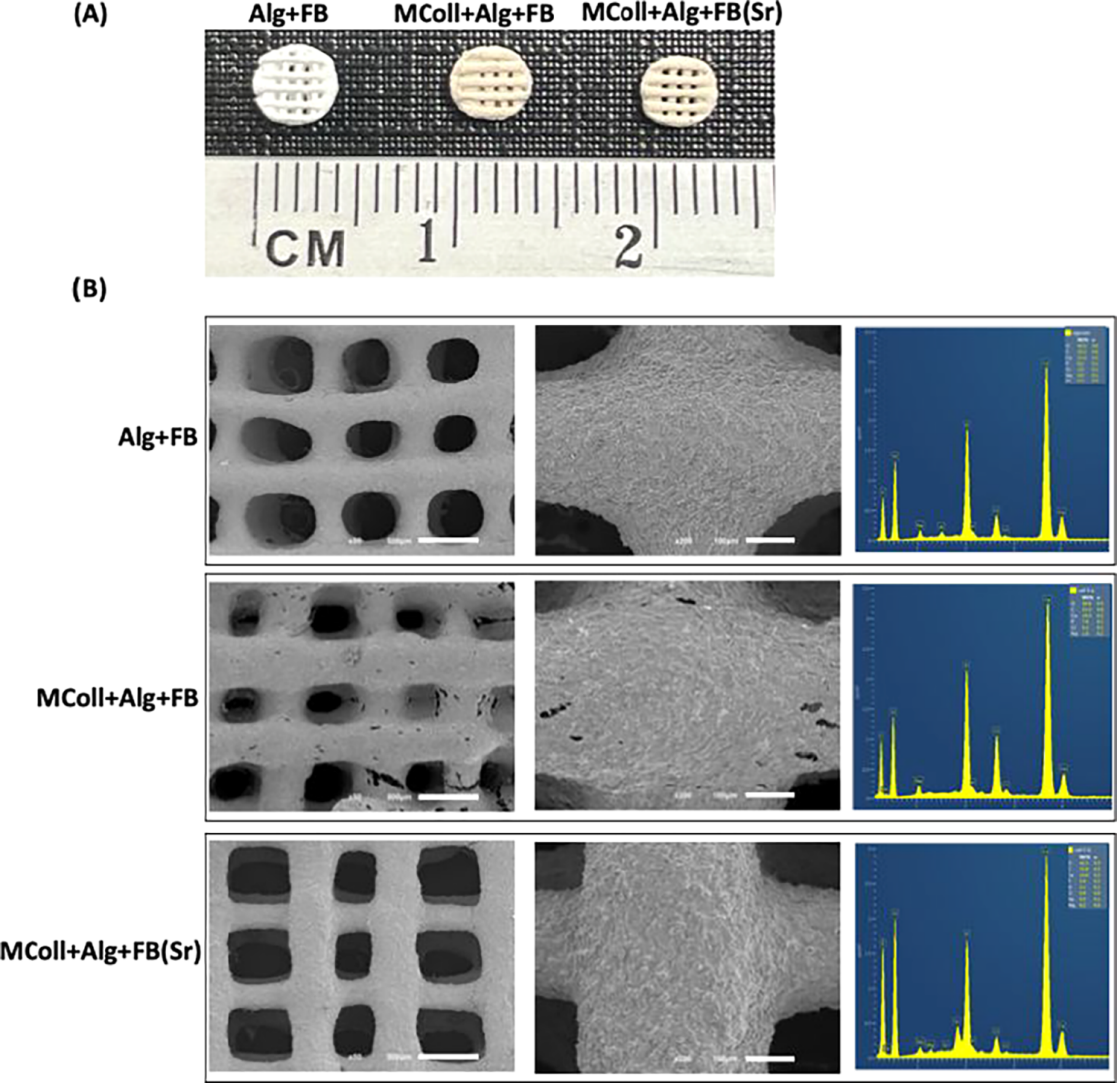
(A) Macroscopic images of the developed 3D-printed scaffolds. (B) Scanning electron microscopy (SEM) micrographs and EDS elemental analysis of the developed 3D-printed scaffolds.

The morphological and morphometric analysis of the 3D-printed scaffolds was assessed through μCT (Figure 4). From 3D reconstruction images, a homogeneous distribution of FB/FB­(Sr) (red color) enclosed by the MColl/Alg matrix (green color) was observed, indicating an optimized method of ink preparation and scaffold 3D printing. The mean porosity, interconnectivity, and pore size of the scaffolds are presented in Figure 4B. It was observed that the scaffolds presented similar porosity, interconnectivity, and pore sizes, indicating that printing fidelity was maintained. Considering that to promote bone cell adhesion, migration, and proliferation, a minimum pore size of around 100 μm and good interconnectivity are required, the values obtained appeared to be encouraging for cellular studies.^56^ Additionally, the presence of small pores enhanced the surface area and the constructs’ permeability, thus providing more protein adsorption sites. In the case of bone regeneration applications, their presence can also act as nucleation sites for the precipitation of bone-like apatite, which promotes the expression of osteogenic-related markers.^57^ Furthermore, it is known that structures presenting high porosity facilitate bone ingrowth as it is required for cell migration, nutrient transport, and waste removal, although it can weaken the mechanical properties of the scaffolds.^58^

**Figure 4:**
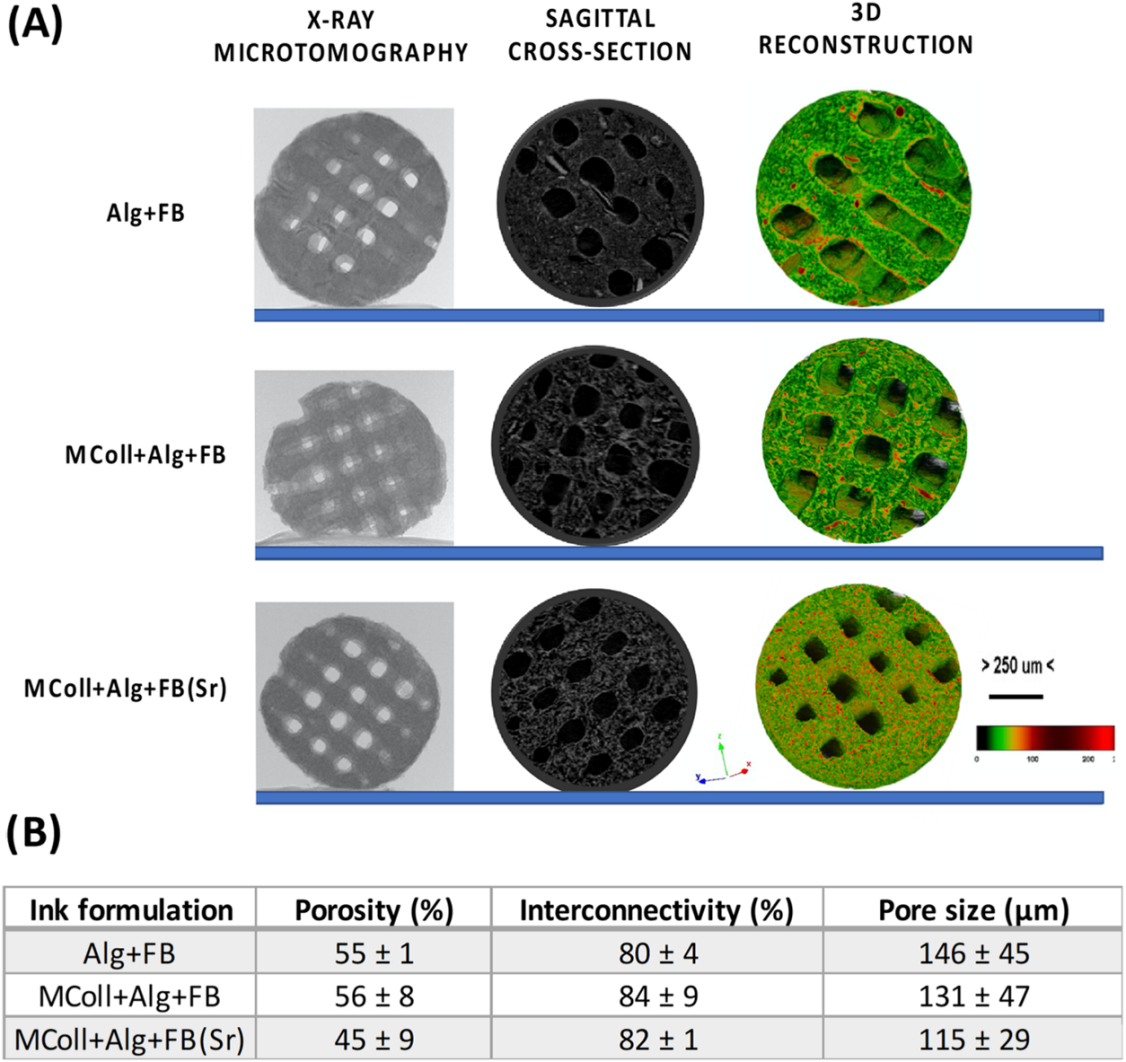
Microcomputed tomography (μCT) analysis of the 3D-printed scaffolds. (A) Representative 2D images and 3D reconstructions of the acquired structure. A uniform distribution of the materials is observed, as indicated by the color scale: green, soft material (MColl and Alg); red, hard material [FB or FB­(Sr)]. (B) Microarchitectural features of the different 3D-printed scaffolds. Data are presented as the mean ± the standard error from three independent experiments.

Therefore, the mechanical properties of the developed 3D-printed scaffolds were evaluated by determining their compressive moduli (Figure 5). Alg-FB scaffolds presented the highest compressive modulus of ∼25 MPa, followed by MColl + Alg + FB­(Sr) and MColl + Alg + FB. Considering the collagen-based scaffolds, the MColl + Alg + FB­(Sr) formulation presented twice the compressive modulus of the undoped formulation. This result is in agreement with the rheological analysis of the ink formulations and suggests that the effect of the ionic cross-linking of Alg with Ca^2+^ also occurs within adjacent printed layers, thus rendering more cohesive structures.^48^ Furthermore, the observed tendency of MColl + Alg + FB­(Sr) scaffolds presenting higher mechanical properties than MColl + Alg + FB has been attributed to more cations (Sr^2+^) being available for the alginate ionic cross-linking process to occur,^48^ although potentially the microscopic fissures observed by SEM on the MColl + Alg + FB formulation have played a detrimental effect on the scaffolds’ mechanical properties. These findings indicate that the Alg + FB and MColl + Alg + FB­(Sr) compositions were within the range of the compressive modulus of human trabecular bone, ranging between 10 and 3000 MPa, depending on the anatomic site and density.^59^ When comparing with the compressive modulus of 3D-printed collagen/chitosan scaffolds and 3D-printed collagen/heparin sulfate scaffolds developed for spinal cord injury, the present results were a considerable improvement.^60,61^ In fact, even mineralized collagen/alginate/silica 3D-printed scaffolds produced for hard tissue regeneration presented lower mechanical properties than the MColl + Alg + FB­(Sr) formulation produced in the current study.^54^ Moreover, the results are in line with photocurable methacrylated silk fibroin 3D-printed hydrogels, produced with low polymer concentration and designed for bone tissue engineering.^62^

**Figure 5:**
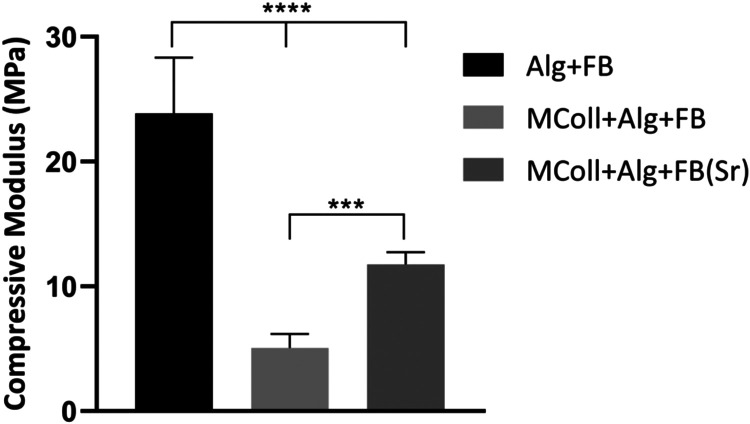
Compressive modulus (Mpa) of the developed 3D-printed scaffolds (*n* = 7, statistical significance for *** *p* ≤ 0.001 and **** *p* ⩽ 0.0001).

### 3.4 *In Vitro* Performance of the 3D-Printed Scaffolds

The applicability of the developed scaffolds as 3D templates for the culture of osteoblast-like cells was assessed *in vitro* using Saos-2 cells, evaluating cell viability and proliferation by Alamar blue and DNA quantification assays, respectively. Cells seeded on the 3D-printed scaffolds were maintained for 14 days without any exogenous stimulation in order to more accurately replicate the microenvironment of the native tissue. The Alamar blue assay and DNA quantification results are depicted in Figure 6A,B, respectively.

**Figure 6:**
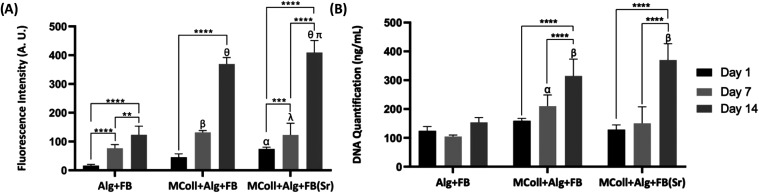
*In vitro* assessment of the developed 3D-printed scaffolds’ biocompatibility using Saos-2 cell line. (A) Metabolic activity of Saos-2 cells cultured in the scaffolds at 1, 7, and 14 days was determined by Alamar blue assay. Data are mean ± standard error (*n* = 3, statistical significance for *p < 0.05; ** *p* < 0.01; *** *p* < 0.001; and **** *p* < 0.0001, and symbols represent statistical differences: α (****) compared with day 1 of Alg + FB, β (****) and λ (**) compared with day 7 of Alg + FB, θ (****) compared with day 14 of Alg + FB, and π (*) compared with day 14 of MColl + Alg + FB). (B) DNA quantification of Saos-2 cells cultured in the scaffolds at 1, 7, and 14 days after cell seeding. Data are mean ± standard error (*n* = 3, statistical significance for **** *p* < 0.0001, and symbols represent statistical differences: α (****) compared with day 7 of Alg + FB and β (****) compared with day 14 of Alg + FB).

An increase in the cell metabolic activity over culturing time could be observed in all formulations (Figure 6 A). However, the collagen-based formulations displayed the best biological performance, as MColl + Alg + FB­(Sr) scaffold presented the highest cell metabolic activity followed by MColl + Alg + FB, which was most pronounced on day 14. Although the noncytotoxicity of CaPs obtained from several fish bones had been previously demonstrated,^19,44^ herein it was shown the beneficial effect of Sr-doping on cell metabolism, in accordance with previous studies.^36,37^ Furthermore, the combination of collagen and CaPs had been found to improve cell viability when comparing with scaffolds constituted by only one of the materials.^63,64^ Additionally, one of the few studies employing marine collagen to develop 3D-printed scaffolds for tissue engineering only maintained cells for 7 days, presenting a more modest increase in cell metabolism than the present study, supporting the enhancement of cellular performance attributed to the presence of CaPs in the respective scaffolds.^65^

Moreover, a significant increase in cell number was observed up to 14 days of culture on the collagen-based scaffolds, as determined by DNA quantification (Figure 6 B). MColl + Alg + FB­(Sr) scaffolds presented the highest cell proliferation values at day 14, although both collagen-containing formulations displayed the same statistically significant difference in relation to Alg + FB scaffolds at that time point. Both marine-origin CaPs and collagen are known to enhance cell proliferation on developed scaffolds,^15,66^ and these results further demonstrate that both *C. reniformis* collagen and Sr-doped CaPs were beneficial for Saos-2 metabolic activity and proliferation.

The alkaline phosphatase (ALP) activity of exposed cells in basal medium to the scaffolds is presented in Figure 7. ALP is highly expressed in mineralized tissue cells and has a pivotal role in hard tissue formation, namely in the calcification of bones.^67^ It enhances mineralization by increasing inorganic phosphate local rates and decreases the inhibition of mineral formation by reducing the extracellular pyrophosphate concentration.^67^ Although the Saos-2 cell line expresses high ALP activity, it is possible to determine its activity over time. In that sense, it can be observed an increase of ALP activity over time in all formulations, indicating that the cells preserved their mineralization capacity under all conditions (Figure 7). Remarkably, at day 1, the cells seeded onto MColl + Alg + FB­(Sr) scaffolds presented the lowest ALP activity of all formulations, and at day 7, their ALP activity levels were still statistically significantly lower than in the MColl + Alg + FB scaffolds. However, at day 14, ALP activity was substantially higher in both collagen-containing formulations, apparently slightly superior to the MColl + Alg + FB­(Sr) scaffolds. It is well-known that the presence of Sr increases osteoblast proliferation, differentiation, and survival, indicated by an increased ALP expression.^68^ Additionally, when administered locally, Sr can be very effective, significantly improving the osseointegration of bone implants with fewer side effects than systemic administration.^69^ Moreover, although increased cell proliferation may lead to enhanced values of metabolic and ALP activity, it is evident that the collagen-containing formulations significantly improved the overall biological performance of the scaffolds. Previous works employing both mammalian and marine-origin collagen for the development of 3D-printed scaffolds have demonstrated the potential to stimulate ALP activity over time.^38,63,70,71^ Additionally, *C. reniformis* collagen, which had been previously employed in the development of skin-regeneration applications,^72^ was herein successfully employed for the first time in a 3D-printed scaffold aiming at bone regeneration. Taking this into account, it was concluded that Saos-2 ALP activity was enhanced in the presence of *C. reniformis* collagen and FB­(Sr). Both natural materials were a valuable supplement for the developed structures, improving the mineralization and calcification rates and consequently the scaffolds’ therapeutic potential for hard tissue regeneration therapies.

**Figure 7:**
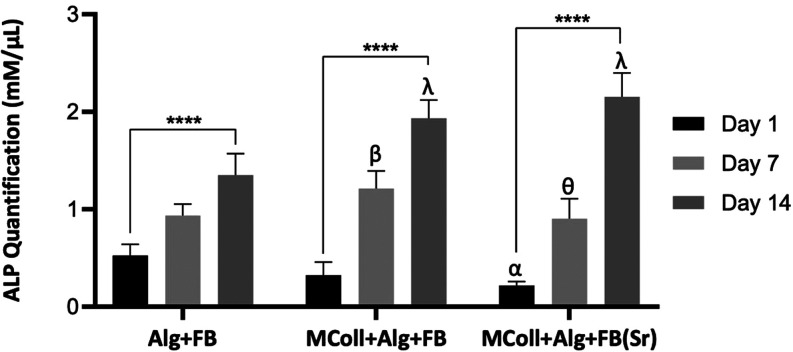
ALP activity of Saos-2 cells cultured on scaffolds for 1, 7, and 14 days. Data are mean ± standard error (*n* = 3, statistical significance for *p < 0.05; ** *p* < 0.01; and **** *p* < 0.0001, and symbols represent statistical differences: α (**) compared with day 1 of Alg + FB, β (*) compared with day 7 of Alg+FB, θ (**) compared with day 7 of MColl + Alg + FB, and λ (****) compared with day 14 of Alg + FB).

The live/dead assay demonstrated that all of the 3D-printed scaffolds developed in this study were noncytotoxic, in line with DNA quantification results (Figure 8). At day 1, the results were similar for all formulations, as most cells were viable, demonstrating the successful cell seeding procedure. In addition, there was a pronounced increase in the number of viable cells at days 7 and 14 in the collagen-based formulations. These qualitative results corroborated the quantitative results presented above, demonstrating the ability of the collagen-based 3D-printed scaffolds, particularly MColl + Alg + FB­(Sr), to support osteoblast-like cell culture while indicating that the alginate-based formulation did not provide the required cues for cells to adhere and proliferate, as previously reported elsewhere.^73^

**Figure 8:**
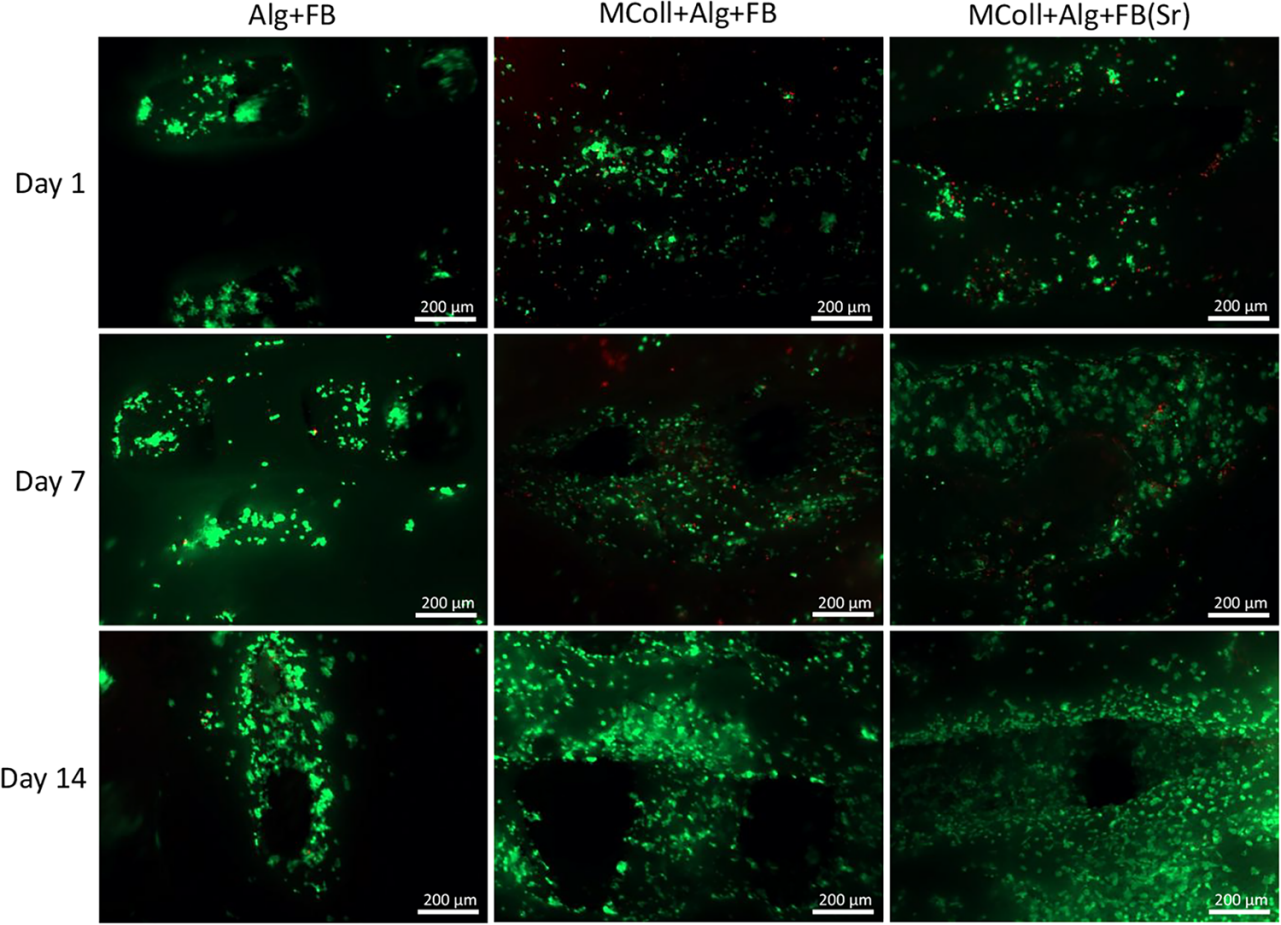
Microscopic images demonstrating the viability of Saos-2 cells on the different 3D-printed scaffolds after 1, 7, and 14 days of culturing. Viable cells are indicated by the green color (stained with calcein-AM), and dead cells are indicated by the red color (stained with PI). Scale bar: 200 μm.

SEM images of the cells seeded on the 3D-printed scaffolds are presented in Figure 9. It was possible to observe cell proliferation over time in the collagen-containing formulations, in agreement with the previously obtained results. While MColl + Alg + FB scaffolds supported both cell proliferation and cell spreading, they were more evident and pronounced in the MColl + Alg + FB­(Sr) scaffolds after 14 days of culturing. This further demonstrated the positive effect of *C. reniformis* collagen^8,72^ and reinforced the beneficial effect of FB­(Sr) on osteoblast-like cells.^33^

**Figure 9:**
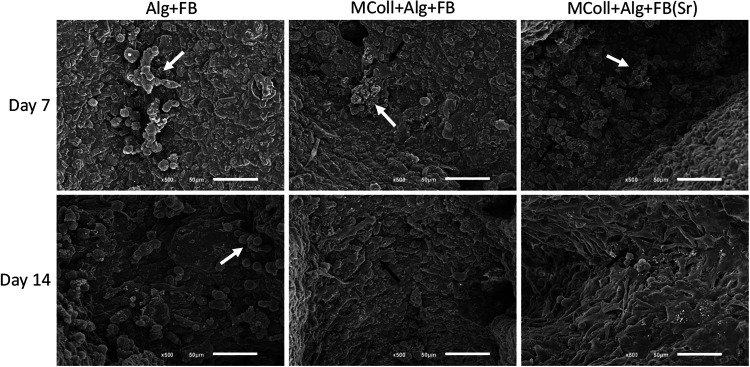
SEM micrographs of the Saos-2 cells cultured onto the 3D-printed scaffolds at 7 and 14 days. Scale bar: 50 μm. White arrows indicate round cells, and black arrows indicate elongated cells.

Although various reports of collagen-based scaffolds, with or without CaPs, aiming at bone regeneration have been published,^54,74^ this is the first work to develop *C. reniformis* collagen-based 3D-printed scaffolds for that purpose. In line with other marine collagens that have been successfully employed to develop hard tissue regeneration therapies, we demonstrated the feasibility of another sustainable collagen source for this specific application.^47,75,76^ Furthermore, the biological performance of MColl + Alg + FB­(Sr) scaffolds, namely regarding cell proliferation and ALP activity, was superior to formulations containing synthetic polymers or collagen chemical cross-linking agents reported in the literature.^38,77^ Therefore, this study serves as a proof-of-concept for the development of 3D-printed cell-instructive scaffolds based on marine-derived collagen for bone regeneration.

## 4 Conclusions

Although collagen is recognized as a highly valuable material for biomedicine, namely, in biomaterials aiming tissue regeneration, its use for 3D printing applications has been hampered by particular requirements, namely, regarding the concentration of the collagen solution and printing parameters. Nevertheless, smart solutions are being developed to overcome these issues, and in the present work, novel 3D-printed scaffolds using blends of alginate and *C. reniformis* collagen combined with Sr-doped CaPs obtained from codfish bones were successfully achieved, aiming for bone tissue engineering applications. The scaffolds presented an appropriate pore size and porosity, along with high interconnectivity, possessing adequate mechanical properties for bone tissue engineering, matching those exhibited by cancellous bone. The *in vitro* assays using Saos-2 cells cultured on the scaffolds disclosed enhanced cell viability and proliferation, more notorious for the case of scaffolds containing Sr., Therefore, this proof-of-concept study demonstrates the manufacturing of biomaterials derived entirely from sustainable marine sources, inspired in the blue economy concept, and advocates for innovation in the health sector. In particular, *C. reniformis* and codfish bones can be used as sustainable sources of collagen and natural calcium phosphates to be used in biomedicine, with the originated 3D-printed structures demonstrating promising *in vitro* performance as bone tissue engineering scaffolds.

For the follow-up of this research line, the incorporation of primary cells is planned to comprehensively evaluate phenotype maintenance and osteogenic differentiation. Furthermore, the production of bioinks is envisioned given the cell-friendly nature of the ink production process.

## References

[1] Lin K., Zhang D., Macedo M. H., Cui W., Sarmento B., Shen G. (2019). Advanced Collagen-Based Biomaterials for Regenerative Biomedicine. Adv. Funct. Mater..

[2] Schlie-Wolter S., Ngezahayo A., Chichkov B. N. (2013). The Selective Role of ECM Components
on Cell Adhesion, Morphology, Proliferation and Communication in Vitro. Exp. Cell Res..

[3] Easterbrook C., Maddern G. (2008). Porcine and Bovine
Surgical Products: Jewish, Muslim,
and Hindu Perspectives. Arch. Surg..

[4] Subhan F., Ikram M., Shehzad A., Ghafoor A. (2015). Marine Collagen: An
Emerging Player in Biomedical Applications. J. Food Sci. Technol..

[5] Fassini D., Wilkie I. C., Pozzolini M., Ferrario C., Sugni M., Rocha M. S., Giovine M., Bonasoro F., Silva T. H., Reis R. L. (2021). Diverse and Productive
Source of Biopolymer Inspiration:
Marine Collagens. Biomacromolecules.

[6] Gökalp M., Wijgerde T., Murk A., Osinga R. (2022). Design for Large-Scale
Maricultures of the Mediterranean Demosponge *Chondrosia
reniformis* Nardo, 1847 for Collagen Production. Aquaculture.

[7] Garrone R., Huc A., Junqua S. (1975). Fine Structure
and Physicochemical Studies on the Collagen
of the Marine Sponge *Chondrosia reniformis* Nardo. J. Ultrasruct. Res..

[8] Rocha M. S., Marques C. F., Carvalho A. C., Martins E., Ereskovsky A., Reis R. L., Silva T. H. (2024). The Characterization
and Cytotoxic
Evaluation of *Chondrosia reniformis* Collagen Isolated from Different Body Parts (Ectosome and Choanosome)
Envisaging the Development of Biomaterials. Mar. Drugs.

[9] Pozzolini M., Scarfì S., Gallus L., Castellano M., Vicini S., Cortese K., Gagliani M. C., Bertolino M., Costa G., Giovine M. (2018). Production, Characterization and
Biocompatibility Evaluation of Collagen Membranes Derived from Marine
Sponge *Chondrosia reniformis* Nardo,
1847. Mar. Drugs.

[10] Swatschek D., Schatton W., Müller W. E. G., Kreuter J. (2002). Microparticles Derived
from Marine Sponge Collagen (SCMPs): Preparation, Characterization
and Suitability for Dermal Delivery of All-Trans Retinol. Eur. J. Pharm. Biopharm..

[11] Wang X., Ao Q., Tian X., Fan J., Wei Y., Hou W., Tong H., Bai S. (2016). Correction:
3D Bioprinting Technologies
for Hard Tissue and Organ Engineering. Materials.

[12] Ng W. L., An J., Chua C. K. (2024). Process,
Material, and Regulatory Considerations for
3D Printed Medical Devices and Tissue Constructs. Engineering.

[13] Zieliński P. S., Gudeti P. K. R., Rikmanspoel T., Włodarczyk-Biegun M. K. (2023). 3D Printing
of Bio-Instructive Materials: Toward Directing the Cell. Bioact. Mater..

[14] Lee J. M., Suen S. K. Q., Ng W. L., Ma W. C., Yeong W. Y. (2021). Bioprinting
of Collagen: Considerations, Potentials, and Applications. Macromol. Biosci..

[15] Yang X., Lu Z., Wu H., Li W., Zheng L., Zhao J. (2018). Collagen-Alginate
as Bioink for Three-Dimensional (3D) Cell Printing Based Cartilage
Tissue Engineering. Mater. Sci. Eng., C.

[16] Inzana J. A., Olvera D., Fuller S. M., Kelly J. P., Graeve O. A., Schwarz E. M., Kates S. L., Awad H. A. (2014). 3D Printing of Composite
Calcium Phosphate and Collagen Scaffolds for Bone Regeneration. Biomaterials.

[17] Terzioğlu P., Öğüt H., Kalemtaş A. (2018). Natural Calcium
Phosphates from Fish Bones and Their Potential Biomedical Applications. Mater. Sci. Eng., C.

[18] Kim S. K., Mendis E. (2006). Bioactive Compounds from Marine Processing
Byproducts
- A Review. Food Res. Int..

[19] Zhu Q., Ablikim Z., Chen T., Cai Q., Xia J., Jiang D., Wang S. (2017). The Preparation and
Characterization
of HA/β-TCP Biphasic Ceramics from Fish Bones. Ceram. Int..

[20] Bouler J. M., Pilet P., Gauthier O., Verron E. (2017). Biphasic Calcium Phosphate
Ceramics for Bone Reconstruction: A Review of Biological Response. Acta Biomater..

[21] Kamitakahara M., Ohtsuki C., Miyazaki T. (2008). Review Paper: Behavior
of Ceramic
Biomaterials Derived from Tricalcium Phosphate in Physiological Condition. J. Biomater. Appl..

[22] Bose S., Fielding G., Tarafder S., Bandyopadhyay A. (2013). Understanding
of Dopant-Induced Osteogenesis and Angiogenesis in Calcium Phosphate
Ceramics. Trends Biotechnol..

[23] Aimaiti A., Maimaitiyiming A., Boyong X., Aji K., Li C., Cui L. (2017). Low-Dose Strontium
Stimulates Osteogenesis but High-Dose Doses Cause
Apoptosis in Human Adipose-Derived Stem Cells via Regulation of the
ERK1/2 Signaling Pathway. Stem Cell Res. Ther..

[24] Robey, P. G. ; Boskey, A. L. The Composition of Bone. In Primer on the Metabolic Bone Diseases and Disorders of Mineral Metabolism, 7th ed.; Wiley, 2008; Vol. 7, pp 32–38.

[25] Gökalp M., Kooistra T., Rocha M. S., Silva T. H., Osinga R., Murk A. J., Wijgerde T. (2020). The Effect
of Depth on the Morphology,
Bacterial Clearance, and Respiration of the Mediterranean Sponge *Chondrosia reniformis* (Nardo, 1847). Mar. Drugs.

[26] Diogo G. S., Marques C. F., Sotelo C. G., Pérez-Martín R. I., Pirraco R. P., Reis R. L., Silva T. H. (2020). Cell-Laden Biomimetically
Mineralized Shark-Skin-Collagen-Based 3D Printed Hydrogels for the
Engineering of Hard Tissues. ACS Biomater. Sci.
Eng..

[27] Dai Z., Ronholm J., Tian Y., Sethi B., Cao X. (2016). Sterilization
Techniques for Biodegradable Scaffolds in Tissue Engineering Applications. J. Tissue Eng..

[28] Martin V., Ribeiro I. A., Alves M. M., Gonçalves L., Claudio R. A., Grenho L., Fernandes M. H., Gomes P., Santos C. F., Bettencourt A. F. (2019). Engineering
a Multifunctional 3D-Printed PLA-Collagen-Minocycline-NanoHydroxyapatite
Scaffold with Combined Antimicrobial and Osteogenic Effects for Bone
Regeneration. Mater. Sci. Eng., C.

[29] Frank-Kamenetskaya O.
V., Rozhdestvenskaya I. V., Rosseeva E. V., Zhuravlev A. V. (2014). Refinement
of Apatite Atomic Structure of Albid Tissue of Late Devon Conodont. Crystallogr. Rep..

[30] Arcos D., Rodríguez-Carvajal J., Vallet-Regí M. (2004). The Effect
of the Silicon Incorporation on the Hydroxylapatite Structure. A Neutron
Diffraction Study. Solid State Sci..

[31] Piccirillo C., Silva M. F., Pullar R. C., Da Cruz I. B., Jorge R., Pintado M. M. E., Castro P. M. L. (2013). Extraction
and Characterisation of
Apatite- and Tricalcium Phosphate-Based Materials from Cod Fish Bones. Mater. Sci. Eng., C.

[32] Bigi A., Boanini E., Capuccini C., Gazzano M. (2007). Strontium-Substituted
Hydroxyapatite Nanocrystals. Inorg. Chim. Acta.

[33] Marques C. F., Olhero S., Abrantes J. C. C., Marote A., Ferreira S., Vieira S. I., Ferreira J. M. F. (2017). Biocompatibility and Antimicrobial
Activity of Biphasic Calcium Phosphate Powders Doped with Metal Ions
for Regenerative Medicine. Ceram. Int..

[34] Goto T., Sasaki K. (2014). Effects of Trace Elements in Fish Bones on Crystal
Characteristics of Hydroxyapatite Obtained by Calcination. Ceram. Int..

[35] Batool S., Hussain Z., Liaqat U., Sohail M. (2022). Solid-State Synthesis
and Process Optimization of Bone Whitlockite. Ceram. Int..

[36] Pal A., Nasker P., Paul S., Chowdhury A. R., Sinha A., Das M. (2019). Strontium Doped Hydroxyapatite
from
Mercenaria Clam Shells: Synthesis, Mechanical and Bioactivity Study. J. Mech. Behav. Biomed. Mater..

[37] Handa S., Nath D., Pal A., Sah M. K. (2024). Biomimetic Hydroxyapatite
and Strontium-Doped Derivatives from Crab Shells and Their Ingenious
Scaffold Fabrication for Bone Tissue Engineering. Mater. Today Commun..

[38] Kim S. C., Heo S. Y., Oh G. W., Yi M., Jung W. K. (2022). A 3D-Printed
Polycaprolactone/Marine Collagen Scaffold Reinforced with Carbonated
Hydroxyapatite from Fish Bones for Bone Regeneration. Mar. Drugs.

[39] Nahar
U K., Shovon B., Chandra D R., Chandra P S., Shukanta B., Muhammed Y. M., Islam MD S. (2017). Characterization of
Beta-Tricalcium Phosphate (β- TCP) Produced at Different Process
Conditions. J. Bioeng. Biomed. Sci..

[40] Mondal S., Mondal A., Mandal N., Mondal B., Mukhopadhyay S. S., Dey A., Singh S. (2014). Physico-Chemical
Characterization and Biological Response
of Labeo Rohita-Derived Hydroxyapatite Scaffold. Bioprocess Biosyst. Eng..

[41] Aydin G., Terzioğlu P., Öğüt H., Kalemtas A. (2021). Production,
Characterization, and Cytotoxicity of Calcium Phosphate Ceramics Derived
from the Bone of Meagre Fish, *Argyrosomus regius*. J. Aust. Ceram. Soc..

[42] Naga S. M., El-Maghraby H. F., Mahmoud E. M., Talaat M. S., Ibrhim A. M. (2015). Preparation
and Characterization of Highly Porous Ceramic Scaffolds Based on Thermally
Treated Fish Bone. Ceram. Int..

[43] Venkatesan J., Lowe B., Manivasagan P., Kang K. H., Chalisserry E. P., Anil S., Kim D. G., Kim S. K. (2015). Isolation and Characterization
of Nano-Hydroxyapatite from Salmon Fish Bone. Materials.

[44] Boutinguiza M., Pou J., Comesaña R., Lusquiños F., de Carlos A., León B. (2012). Biological Hydroxyapatite Obtained
from Fish Bones. Mater. Sci. Eng., C.

[45] Ivankovic H., Tkalcec E., Orlic S., Gallego Ferrer G., Schauperl Z. (2010). Hydroxyapatite Formation from Cuttlefish Bones: Kinetics. J. Mater. Sci. Mater. Med..

[46] Piccirillo C., Pullar R. C., Tobaldi D. M., L Castro P. M., E Pintado M. M. (2014). Hydroxyapatite
and Chloroapatite Derived from Sardine By-Products. Ceram. Int..

[47] Diogo G. S., Marques C. F., Freitas-Ribeiro S., Sotelo C. G., Pérez-Martin R.
I., Pirraco R. P., Reis R. L., Silva T. H. (2022). Mineralized Collagen
as a Bioactive Ink to Support Encapsulation of Human Adipose Stem
Cells: A Step towards the Future of Bone Regeneration. Biomater. Adv..

[48] Aguero L., Alpdagtas S., Ilhan E., Zaldivar-Silva D., Gunduz O. (2021). Functional Role of Crosslinking in Alginate Scaffold
for Drug Delivery and Tissue Engineering: A Review. Eur. Polym. J..

[49] Lin H. R., Yen Y. J. (2004). Porous Alginate/Hydroxyapatite
Composite Scaffolds
for Bone Tissue Engineering: Preparation, Characterization, and in
Vitro Studies. J. Biomed. Mater. Res..

[50] Das S., Pati F., Choi Y. J., Rijal G., Shim J. H., Kim S. W., Ray A. R., Cho D. W., Ghosh S. (2015). Bioprintable,
Cell-Laden Silk Fibroin-Gelatin Hydrogel Supporting Multilineage Differentiation
of Stem Cells for Fabrication of Three-Dimensional Tissue Constructs. Acta Biomater..

[51] Giuseppe M. Di., Law N., Webb B., A Macrae R., Liew L. J., Sercombe T. B., Dilley R. J., Doyle B. J. (2018). Mechanical Behaviour of Alginate-Gelatin
Hydrogels for 3D Bioprinting. J. Mech. Behav.
Biomed. Mater..

[52] Lee J. U., Yeo M., Kim W. J., Koo Y. W., Kim G. H. (2018). Development of a
Tannic Acid Cross-Linking Process for Obtaining 3D Porous Cell-Laden
Collagen Structure. Int. J. Biol. Macromol..

[53] Rhee S., Puetzer J. L., Mason B. N., Reinhart-King C. A., Bonassar L. J. (2016). 3D Bioprinting of Spatially Heterogeneous
Collagen
Constructs for Cartilage Tissue Engineering. ACS Biomater. Sci. Eng..

[54] Lee H., Kim Y., Kim S., Kim G. (2014). Mineralized Biomimetic Collagen/Alginate/Silica
Composite Scaffolds Fabricated by a Low-Temperature Bio-Plotting Process
for Hard Tissue Regeneration: Fabrication, Characterisation and in
Vitro Cellular Activities. J. Mater. Chem. B.

[55] Bell A., Kofron M., Nistor V. (2015). Multiphoton
Crosslinking for Biocompatible
3D Printing of Type I Collagen. Biofabrication.

[56] Jones A. C., Arns C. H., Hutmacher D. W., Milthorpe B. K., Sheppard A. P., Knackstedt M. A. (2009). The Correlation
of Pore Morphology,
Interconnectivity and Physical Properties of 3D Ceramic Scaffolds
with Bone Ingrowth. Biomaterials.

[57] Zhang K., Fan Y., Dunne N., Li X. (2018). Effect of Microporosity on Scaffolds
for Bone Tissue Engineering. Regener. Biomater..

[58] Li B. Q., Wang C. Y., Lu X. (2013). Effect of
Pore Structure on the Compressive
Property of Porous Ti Produced by Powder Metallurgy Technique. Mater. Des..

[59] Morgan E. F., Unnikrisnan G. U., Hussein A. I. (2018). Bone Mechanical
Properties in Healthy
and Diseased States. Annu. Rev. Biomed. Eng..

[60] Sun Y., Yang C., Zhu X., Wang J. J., Liu X. Y., Yang X. P., An X. W., Liang J., Dong H. J., Jiang W., Chen C., Wang Z. G., Sun H. T., Tu Y., Zhang S., Chen F., Li X. H. (2019). 3D Printing Collagen/Chitosan
Scaffold Ameliorated Axon Regeneration and Neurological Recovery after
Spinal Cord Injury. J. Biomed. Mater. Res..

[61] Chen C., Zhao M. L., Zhang R. K., Lu G., Zhao C. Y., Fu F., Sun H. T., Zhang S., Tu Y., Li X. H. (2017). Collagen/Heparin
Sulfate Scaffolds Fabricated by a 3D Bioprinter Improved Mechanical
Properties and Neurological Function after Spinal Cord Injury in Rats. J. Biomed. Mater. Res..

[62] Rajput M., Mondal P., Yadav P., Chatterjee K. (2022). Light-Based
3D Bioprinting of Bone Tissue Scaffolds with Tunable Mechanical Properties
and Architecture from Photocurable Silk Fibroin. Int. J. Biol. Macromol..

[63] Fahimipour F., Dashtimoghadam E., Rasoulianboroujeni M., Yazdimamaghani M., Khoshroo K., Tahriri M., Yadegari A., Gonzalez J. A., Vashaee D., Lobner D. C., Jafarzadeh Kashi T. S., Tayebi L. (2018). Collagenous Matrix Supported by a
3D-Printed Scaffold
for Osteogenic Differentiation of Dental Pulp Cells. Dent. Mater..

[64] Seo S. J., Kim Y. G. (2021). Improved Bone Regeneration Using
Collagen-Coated Biphasic
Calcium Phosphate with High Porosity in a Rabbit Calvarial Model. Biomed. Mater..

[65] Govindharaj M., Roopavath U. K., Rath S. N. (2019). Valorization of
Discarded Marine
Eel Fish Skin for Collagen Extraction as a 3D Printable Blue Biomaterial
for Tissue Engineering. J. Cleaner Prod..

[66] Bas M., Daglilar S., Kuskonmaz N., Kalkandelen C., Erdemir G., Kuruca S. E., Tulyaganov D., Yoshioka T., Gunduz O., Ficai D., Ficai A. (2020). Mechanical
and Biocompatibility Properties of Calcium Phosphate Bioceramics Derived
from Salmon Fish Bone Wastes. Int. J. Mol. Sci..

[67] Vimalraj S. (2020). Alkaline Phosphatase:
Structure, Expression and Its Function in Bone Mineralization. Gene.

[68] Marx D., Yazdi A. R., Papini M., Towler M. (2020). A Review of the Latest
Insights into the Mechanism of Action of Strontium in Bone. Bone Rep..

[69] Kołodziejska B., Stępień N., Kolmas J. (2021). The Influence
of Strontium
on Bone Tissue Metabolism and Its Application in Osteoporosis Treatment. Int. J. Mol. Sci..

[70] Lin K. F., He S., Song Y., Wang C. M., Gao Y., Li J. Q., Tang P., Wang Z., Bi L., Pei G. X. (2016). Low-Temperature
Additive Manufacturing of Biomimic Three-Dimensional Hydroxyapatite/Collagen
Scaffolds for Bone Regeneration. ACS Appl. Mater.
Interfaces.

[71] Elango J., Zhang J., Bao B., Palaniyandi K., Wang S., Wenhui W., Robinson J. S. (2016). Rheological, Biocompatibility
and Osteogenesis Assessment of Fish Collagen Scaffold for Bone Tissue
Engineering. Int. J. Biol. Macromol..

[72] Tassara E., Oliveri C., Vezzulli L., Cerrano C., Xiao L., Giovine M., Pozzolini M. (2023). 2D Collagen
Membranes from Marine
Demosponge *Chondrosia reniformis* (Nardo,
1847) for Skin-Regenerative Medicine Applications: An In Vitro Evaluation. Mar. Drugs.

[73] Rowley J. A., Madlambayan G., Mooney D. J. (1999). Alginate Hydrogels as Synthetic Extracellular
Matrix Materials. Biomaterials.

[74] Levingstone T. J., Thompson E., Matsiko A., Schepens A., Gleeson J. P., O’brien F. J. (2016). Multi-Layered
Collagen-Based Scaffolds for Osteochondral
Defect Repair in Rabbits. Acta Biomater..

[75] Diogo G. S., López-Senra E., Pirraco R. P., Canadas R. F., Fernandes E. M., Serra J., Pérez-Martín R. I., Sotelo C. G., Marques A. P., González P., Moreira-Silva J., Silva T. H., Reis R. L. (2018). Marine Collagen/Apatite Composite
Scaffolds Envisaging Hard Tissue Applications. Mar. Drugs.

[76] Hoyer B., Bernhardt A., Heinemann S., Stachel I., Meyer M., Gelinsky M. (2012). Biomimetically
Mineralized Salmon Collagen Scaffolds
for Application in Bone Tissue Engineering. Biomacromolecules.

[77] Kim W. J., Jang C. H., Kim G. H. (2017). Optimally
Designed Collagen/Polycaprolactone
Biocomposites Supplemented with Controlled Release of HA/TCP/RhBMP-2
and HA/TCP/PRP for Hard Tissue Regeneration. Mater. Sci. Eng., C.

